# Supplementary material for: HIV self-testing awareness among African refugee male sex workers in Italy: A mixed-methods study
Source: PLoS One. 2026 Feb 23;21(2):e0343441. doi: 10.1371/journal.pone.0343441 (PMC12928482; doi:10.1371/journal.pone.0343441)
Supplement: S1 Table — (DOCX) [file pone.0343441.s001.docx]

### Table S1 Study variables and coding scheme

| Variable | Description | Coding |
| --- | --- | --- |
| **Primary Outcomes** | | |
| Awareness of HIVST | Ever heard of an HIV self-test | 1 = Yes, 0 = No |
| Socio-Demographic & Structural Factors | | |
| Age | Participant's age | 0 = 18-24, 1 = 25+ |
| Gender Identity | Self-identified gender | 1 = Man  2 = Transgender  3 = non-binary |
| Education | Highest level of education completed | 1 = Primary or less  2 = Secondary or more |
| Marital Status | Current marital status | 0 = Unmarried  1 = Married |
| Number of Children | Number of children | 0 = No children  1= One or more children |
| Religious Affiliation | Whether the participant belongs to any religion | 0 = No religion  1 = Belong to a religion |
| Rank of Religiosity | How religious the participant is | 0 = Not religious  1 = Religious |
| Length of Stay in Italy | How long participant has been in Italy | 0 = A year or less  1 = More than a year |
| Sexual Orientation | Participant’s sexual orientation | 0 = Gay  1 = Bisexual |
| Sex role | Sexual role preference | 0 = Top  1 = Bottom  2= Versatile (Verse) |
| Employment Beyond Sex Work | Any other job apart from sex work | 0 = No  1 = Yes |
| **Healthcare Access & Testing Behavior** | | |
| Distance to Healthcare Facility (Km) | Distance in kilometers | 0 = 1 – 5km  1 = 6km or more |
| Healthcare frequency | Frequency of accessing healthcare | 0 = Never  1 = Have ever |
| Health Insurance status | Covered by health insurance | 0 = No  1 = Yes |
| Immigration challenge in healthcare access | Encountered difficulties in access healthcare due to immigration status | 0 = No  1 = Yes |
| Experienced stigma or discrimination at the hospital | Experienced stigma or discrimination at the hospital | 0 = No  1 = Yes |
| Awareness of testing facilities | Do you know where to go for STI/HIV testing and care? | 0 = No  1 = Yes |
| Tested for STI | Ever tested for any STI | 0 = Never tested  1 = Ever tested |
| Tested for HIV | Ever tested for HIV | 0 = Never tested  1 = Ever tested |
| HIV test frequency | Frequency of HIV testing | 0 = Once a year or less  1 = More than once a year |
| HIV test result | Self-reported HIV test result | 0 = Negative  1 = Positive |
| Awareness of HIVST | Ever heard of HIV self-testing | 0 = No  1 = Yes |
| Ever used HIVST | Used an HIV self-test before | 0 = No  1 = Yes |
| **Sex Work-Related Factors** | | |
| Venue for meeting client | Where participants find clients | 0 = Offline  1 = Online |
| No. of client in past 2month (Men) | Number of male clients in the past 2 months | 0 = Less than 20  1 = 20 or more |
| No. of client in past 2 month (Women) | Number of female clients in the past 2 months | 0 = Less than 10  1 = 10 or more |
| Vaginal sex | Engaged in vaginal sex | 0 = No  1 = Yes |
| Condom use for vaginal sex | Consistency of condom use for vaginal sex | 0 = Sometimes/Never  1 = Always |
| Anal sex | Engaged in anal sex | 0 = No  1 = Yes |
| Condom use for anal sex | Consistency of condom use for anal sex | 0 = Sometimes/Never  1 = Always |
| Lubricant use for anal sex | Consistency of lubricant use for anal sex | 0 = Sometimes/Never  1 = Always |
| Transactional condomless sex | Engaged in unprotected transactional sex | 0 = No  1 = Yes |
