# Supplementary material for: HIV self-testing awareness among African refugee male sex workers in Italy: A mixed-methods study
Source: PLoS One. 2026 Feb 23;21(2):e0343441. doi: 10.1371/journal.pone.0343441 (PMC12928482; doi:10.1371/journal.pone.0343441)
Supplement: S2 Table — (DOCX) [file pone.0343441.s002.docx]

**Table S2 Country of Origin - Full Breakdown)**

| **Country** | **N (%)** |
| --- | --- |
| Nigeria | 86 (57.3) |
| Cameroon | 6 (4.0) |
| Ghana | 16 (10.7) |
| Angola | 4 (2.7) |
| Burkina Faso | 3 (2.0) |
| Central African Republic | 2 (1.3) |
| Chad | 5 (3.3) |
| Comoros | 1 (0.7) |
| Congo DR | 4 (2.7) |
| Côte d'Ivoire | 1 (0.7) |
| Gabon | 1 (0.7) |
| The Gambia | 1 (0.7) |
| Guinea | 2 (1.3) |
| Kenya | 1 (0.7) |
| Liberia | 1 (0.7) |
| Benin | 1 (0.7) |
| Mali | 4 (2.7) |
| Namibia | 1 (0.7) |
| Senegal | 6 (4.0) |
| Sudan | 1 (0.7) |
| Togo | 3 (2.0) |
