# Supplementary material for: HIV self-testing awareness among African refugee male sex workers in Italy: A mixed-methods study
Source: PLoS One. 2026 Feb 23;21(2):e0343441. doi: 10.1371/journal.pone.0343441 (PMC12928482; doi:10.1371/journal.pone.0343441)
Supplement: Attachment — Submitted filename: Response to reviewers.doc [file pone.0343441.s003.doc]

**Gamji Rabiu Abu-Ba'are, PhD**

School of Nursing
University of Rochester
Rochester, New York, USA

June 10, 2025

**Editor-in-Chief**
PLOS ONE

Dear Editor,

We would like to thank you and the reviewers for your careful consideration of our manuscript entitled: **"Yes, I Saw It at a Client’s House": Factors Associated with Oral HIV Self-Testing Awareness Among African Refugee Male Sex Workers in Italy. A Mixed-Methods Study - BGSH-022**

We appreciate the thoughtful and constructive feedback provided, which has significantly improved the clarity, depth, and rigor of our work. Below, we provide a detailed response to each of the editorial and reviewer comments. Changes have been made throughout the manuscript accordingly.

**Editorial Comments**

**Editorial Comment 1:** Please ensure that your manuscript meets PLOS ONE's style requirements, including those for file naming.

**Response:** The revised manuscript has been formatted according to PLOS ONE’s style guidelines, including title page formatting, reference style, figure captions, and file naming.

**Editorial Comment 2:** Please include a complete copy of PLOS’ inclusivity in global research questionnaire.

**Response:** We have completed the PLOS Inclusivity Questionnaire and uploaded it as Supporting Information.

**Editorial Comment 3:** Please provide an amended funding statement declaring all funding and include: “There was no additional external funding received for this study.”

**Response:** The Funding Statement has been revised as follows:

This project was supported by award number P30MH062294 from the National Institute of Mental Health (NIMH), awarded to GRA ([https://www.nimh.nih.gov](https://www.nimh.nih.gov/)). The content is solely the responsibility of the authors and does not necessarily represent the official views of the Center for Interdisciplinary Research on AIDS, the National Institute of Mental Health, or the National Institutes of Health. There was no additional external funding received for this study.

**Editorial Comment 4:** Please clarify your data sharing statement.

**Response:** We are unable to publicly share the full dataset due to ethical restrictions imposed by the National Ethics Committee for Clinical Trials (Italian Ministry of Health), as the dataset includes sensitive information from a highly vulnerable population that could compromise participant confidentiality even after de-identification. However, de-identified data may be made available upon reasonable request.

### **ADDITIONAL EDITOR COMMENTS**

**Comment:** Explain how participants were recruited. Add "sample and sampling" subsection.

**Response:** We have added a dedicated "Sample and Sampling" subsection to the Methods section, clearly describing our peer-referral recruitment process, the role of the peer-research assistants, and the rationale for the sample sizes for both the quantitative and qualitative phases. As recruitment was informal, non-incentivized, and without fixed quotas, a flow diagram could not be created due to the absence of detailed referral tracking.

**Comment:** Flowchart and map suggested.

**Response:** As noted, a formal flow diagram could not be constructed due to the informal, peer-driven nature of sampling and absence of recruitment tracking. We have clarified this limitation directly in the manuscript. Unfortunately, we were unable to produce a map as suggested due to the sensitive nature of the population and data limitations. We clarified that recruitment took place in Verona and Turin and explained the sampling limitations in the Methods and Limitations sections

**Comment:** Use PLOS checklists for mixed-method studies.

**Response:** We have completed and uploaded the STROBE checklist (for quantitative reporting). Since qualitative components were also included, we have additionally completed the COREQ checklist and uploaded it as supplementary material.

**Comment:** Revise tables, follow PLOS formatting.

**Response:** All tables and figures have been reformatted according to PLOS submission guidelines.

### **REVIEWER 1 COMMENTS**

**Comment 1:** Simplify the title. Remove “Yes, I saw it at a client’s house” and “BSGH-22”.

**Response:** We have simplified the title to: **Factors Associated with Oral HIV Self-Testing Awareness Among African Refugee Male Sex Workers in Italy. A Mixed-Methods Study.**

**Comment 2:** Introduction too long; focus on objectives.

**Response:** The Introduction has been significantly shortened and refocused to highlight the research gap concerning HIVST awareness among African refugee male sex workers in Europe. We added a brief review of existing European literature and emphasized the lack of data specific to this population in Italy.

**Comment 3:** Better describe snowball sampling and justify sample sizes for quantitative and qualitative components.

**Response:** We have elaborated on our sampling strategy, including how seeds were identified, the peer-led recruitment process, the informal referral structure, and lack of incentive-based recruitment. Sample size decisions were based on prior studies with comparable marginalized populations, logistical feasibility, and available peer networks. Citations of comparable studies have been added.

**Comment 4a:** Discuss education level and HIVST practicability among African sex workers.

**Response:** The Discussion section now includes additional literature demonstrating how lower education levels may impair HIVST usability due to literacy barriers, and how peer support and visual instructions may improve uptake in these groups.

**Comment 4b:**Discuss better the selection bias and generalizability.

**Response:** We expanded the Strengths and Limitations section to acknowledge potential selection bias due to non-probability sampling, referral bias, homophily within peer networks, and limited generalizability beyond the two study cities or broader European contexts.

### **REVIEWER 2 COMMENTS**

**Major Comment 1:** Revise title for conciseness.

**Response:** Addressed above (see Reviewer 1, Comment 1).

**Major Comment 2:**Revise referencing to Vancouver style.

**Response:** All references have been reformatted in Vancouver style, in-text and in reference list, following PLOS submission guidelines.

**Major Comment 3:** Use most recent UNAIDS data; consider alternative source on migrants.

**Response:** We replaced the UNAIDS citation with the suggested ECDC report:
HIV and migrants in the EU/EEA, 2024.

**Major Comment 4:** Clarify how HTS is delivered in Italy.

**Response:** The Introduction has been updated to accurately distinguish between CITC (client-initiated testing), PITC (provider-initiated testing), facility-based, community-based, and HIVST. We also clarified that CITC is most common in Italy, while African refugees may have limited access to facility-based testing.

**Major Comment 5:** Please clarify whether participants used HIVST in Italy or country of origin.

**Response:** We clarified in the text that participants who reported HIVST use were referring to self-testing experiences in Italy. We did not collect data on HIVST use in their country of origin, and this is acknowledged as a limitation.

**Major Comment 6:** Clearly describe qualitative analysis method.

**Response:** We clarified that we employed summative content analysis for qualitative data, following an iterative coding and theme development process. This is now clearly described in the Methods section.

**Major Comment 7:** Revise Results to remove duplications and clarify tables.

**Response:** Results have been streamlined to eliminate duplication. Overlapping tables were merged or moved to appendices. The revised tables are now clearer and consistent with journal requirements.

**Major Comment 8:** Add strengths and weaknesses in Discussion.

**Response:** The Discussion now contains a dedicated "Strengths and Limitations" subsection summarizing both the strengths (e.g., rare access to hidden population, mixed-methods approach, peer recruitment) and weaknesses (e.g., selection bias, non-generalizability, cross-sectional design, lack of referral tracking).

**Major Comment 9:** Revise participant quotations to make each unique (e.g., FGD participant 2).

**Response:** All qualitative quotations have been updated to assign unique identifiers (e.g., IDI Participant 1; FGD Participant 2) to ensure clarity and uniqueness.

**Comment on Peer Review History:**
We agree to allow the peer review history to be published alongside the accepted article.

We sincerely thank the Editor and Reviewers for their detailed comments and valuable suggestions. We believe these comprehensive revisions have substantially strengthened our manuscript.

Sincerely,

Gamji Rabiu Abu-Ba'are, PhD
