# Supplementary material for: HIV self-testing awareness among African refugee male sex workers in Italy: A mixed-methods study
Source: PLoS One. 2026 Feb 23;21(2):e0343441. doi: 10.1371/journal.pone.0343441 (PMC12928482; doi:10.1371/journal.pone.0343441)
Supplement: Attachment — Submitted filename: Response to Reviewers_PLOS ONE 8_01_26.docx [file pone.0343441.s004.docx]

**Response to Reviewers**

**Manuscript ID:** PONE-D-25-61075

**Title:** HIV Self-Testing Awareness Among African Refugee Male Sex Workers in Italy

We thank the Academic Editor and Reviewers for their careful review and constructive feedback. We have revised the manuscript accordingly and believe that these revisions have strengthened the clarity, rigor, and contribution of the study. Below, we provide a point-by-point response to all comments raised.

**Academic Editor Comments**

**Comment 1:** The major weakness of this article is the use of non-probability sampling for the quantitative survey phase. The authors should specify how the validity of the chi-square tests was verified and how confounding factors were controlled.

**Response**: We thank the editor for this important comment. We have revised the Sample and Sampling section to clearly describe the peer-led venue-based and snowball sampling strategy and to justify why respondent-driven sampling (RDS) was not feasible in this context, considering ethical, safety, and logistical constraints among a highly vulnerable refugee population. Additionally, we expanded the Data Analysis section to specify that chi-square test assumptions were systematically assessed, with Fisher’s exact tests applied where assumptions were violated. We also clarified the specification of the multivariable logistic regression model, including adjustment for potential confounders and assessment of multicollinearity using variance inflation factors (VIFs).

**Reviewer #1 Comments**

**Comment 1:** Please revise your title to make it punchier and impactful.

**Response:** The manuscript title has been revised to improve clarity and impact. The revised title is now: **“HIV Self-Testing Awareness Among African Refugee Male Sex Workers in Italy.”**

**Comment 2:** Please revise your introduction to make it shorter and focus on research gaps regarding ARMSWs in Europe.

**Response:** The Introduction has been substantially condensed and reorganized to foreground the European context and to emphasize the research gap concerning African refugee male sex workers. Redundant background material was reduced, and the gap is now articulated more clearly and earlier in the section.

**Comment 3:** Please repeat table headers on subsequent pages.

**Response:** Column headers within tables have been formatted to repeat automatically on subsequent pages.

**Comment 4:** Please include information on participants’ income or number of clients, if available.

**Response:** We thank the reviewer for this suggestion. Quantitative data on income or fees charged per client were not collected, as qualitative findings indicated that pricing among African refugee male sex workers is highly variable and negotiated on a case-by-case basis, depending on factors such as client characteristics, venue, duration of the encounter, perceived risk, and participants’ personal preferences. We therefore did not include a single numeric measure of charges per client, as this would not have accurately captured economic vulnerability. This rationale is now explicitly acknowledged in the Study Strengths and Limitations section. Information on number of clients collected and is reported in the Results section to contextualize participants’ vulnerability.

**Comment 5:** Please include a new “Sample and Sampling” subsection.

**Response:** A dedicated *Sample and Sampling* subsection has been added to clearly describe eligibility criteria, recruitment procedures, and the peer-led snowball sampling approach.

**Comment 6:**Please avoid ending the introduction with questions.

**Response:** The Introduction has been revised to end with a narrative statement of the study objectives rather than research questions.

**Comment 7:** Please further condense the discussion and focus on interpretation and implications.

**Response:** The Discussion section has been substantially condensed to focus on interpretation of the key findings, with clearer links to the Introduction and expanded discussion of implications for future research, policy, and clinical practice relevant to ARMSWs in Italy.

**Comment 8:** Please revise references and typos.

**Response:** The reference list has been reviewed for completeness and accuracy, typographical errors were corrected, and formatting was updated to meet journal requirements.

**Comment 9:** Please consider citing additional HIVST studies from Italy and Europe.

**Response:** We thank the reviewer for this suggestion. Additional European HIVST studies from Spain, the Netherlands, and Italy have been incorporated into the Introduction and Discussion to better contextualize the existing evidence base and highlight the persisting research gap for ARMSWs.

**Journal Requirements**

**Ethics and Refugee Considerations**

Additional details were added to clarify that participation was voluntary, participants could opt out at any time without consequences, and non-participants received the same services as participants.

**Funding Disclosure**

The Role of the Funder statement has been added to our cover letter as requested:
*“The funders had no role in study design, data collection and analysis, decision to publish, or preparation of the manuscript.”*

**Data Availability**

The Data Availability Statement has been revised to clarify ethical restrictions on public data sharing and to specify the procedure for requesting de-identified data.

**Supporting Information**

Supplementary tables were removed from the manuscript and uploaded as separate Supporting Information files, with corresponding legends listed after the References section.

We thank the editor and reviewers again for their constructive feedback and hope that the revisions satisfactorily address all comments.
